# A scoping review of interventions addressing gender-based violence in West Africa: examining typologies, delivery mechanisms, outcomes, and stakeholder involvement

**DOI:** 10.1186/s13031-025-00712-x

**Published:** 2025-10-10

**Authors:** Ifunanya Clara Agu, Mahua Das, Rebecca King, Prince Agwu, Chinyere Ojiugo Mbachu

**Affiliations:** 1 Institute of Public Health, University of Nigeria, Enugu, Nigeria; 2 Leeds Institute of Health Sciences, University of Leeds, Leeds, UK; 3 Health Policy Research Group, University of Nigeria, Enugu, Nigeria; 4 Department of Social Work, University of Nigeria, Enugu, Nigeria; 5 Education and Society, University of Dundee, Dundee, UK; 6 Department of Community Medicine, University of Nigeria, Enugu, Nigeria

**Keywords:** Gender-based violence (GBV), Community-based interventions, Facility-based interventions, School-based interventions, Violence prevention, Scoping review, West africa

## Abstract

**Background:**

Gender-based violence (GBV) remains a significant public health and human rights concern globally, with interventions in West Africa designed to address prevention, response, and survivor support. This scoping review aims to synthesize existing evidence on interventions addressing GBV in West Africa, describing their typologies, delivery mechanisms, and outcomes.

**Methods:**

We used two search strings to identify GBV interventions implemented in West Africa and published between January 2010 and March 2025 across five databases, including PubMed, Zendy, Google Scholar, Medline, and ResearchGate. Two independent reviewers screened the titles, abstracts, and full texts of all identified publications for eligibility and inclusion into the study. A total of 25 studies met the inclusion criteria and were included in the study. Data were analysed thematically and presented based on typologies and mechanisms, outcomes using the WHO RESPECT framework, and stakeholders’ involvement in GBV interventions.

**Results:**

The majority of the studies (10) were conducted in Ghana, with six in Nigeria and only a few in other countries, including Niger (2), Senegal (1), Guinea (1), Sierra Leone (1), Côte d’Ivoire (2), and Liberia (1). 42% (11) of the articles focused on various forms of GBV that were not limited to intimate partner violence (IPV), while 39% (10 articles) specifically targeted IPV interventions. Only 19% (5) of these articles discussed interventions for physical violence, notably domestic violence and female genital mutilation/cutting (FGM/C). The intervention strategies included community-based, facility-based, school-based, and media-technology approaches, aimed at diverse groups such as adolescents, households, pregnant women, men, couples, GBV victims, and healthcare providers. Community-based initiatives, like gender dialogue groups and awareness campaigns, were effective in promoting gender-equitable attitudes; however, maintaining long-term behavioural change proved difficult for the males. Facility-based initiatives, involving sexual assault referral centres (SARCs) and therapeutic counselling, enhanced access to medical and psychological care, although challenges remained in survivor follow-ups and the tertiary level of care.

**Conclusion:**

Our review findings highlight the importance of integrating multi-pronged approaches, combining community-based initiatives, institutional capacity-building, technological innovations, economic empowerment, and long-term sustainability efforts to comprehensively tackle GBV. Future interventions should prioritize addressing emotional and psychological violence, focus on schools and male engagement, and explore scalable models for institutional integration.

**Supplementary Information:**

The online version contains supplementary material available at 10.1186/s13031-025-00712-x.

## Background

Gender-based violence (GBV) remains a pervasive global public health issue, affecting individuals across all regions, cultures, and socio-economic backgrounds [1, 2]. Characterized by actions that lead to physical, sexual, or psychological harm, including threats of such actions and the unjust deprivation of freedom [3, 4]. GBV manifests in various forms, including domestic violence, intimate partner violence (IPV), rape, sexual harassment, emotional abuse, child marriage, and harmful traditional practices such as female genital mutilation (FGM), and widowhood practices [3, 4].

GBV significantly constrains women’s autonomy, making them more vulnerable to further violence [1, 5]. This heightened vulnerability can lead to significant negative impacts on their mental health, increasing feelings of fear, anxiety, and depression [6, 7]. Additionally, women facing GBV often experience poor sexual and reproductive health (SRH) outcomes, as they may be less likely to make informed and timely decisions about their bodies and reproductive choices [8]. The cycle of violence and lack of control over their lives not only erodes their self-esteem but also hinders access to necessary health services, perpetuating a cycle of health disparities. Limited access to education and economic opportunities for women and girls further exacerbates their vulnerability to violence [2]. Although GBV disproportionately affects most women and girls, it could also affect men and boys in different ways [9]. Hence, this study will include documented evidence of interventions implemented to address GBV among adult men and women, as well as adolescent girls and boys. Addressing GBV is critical for advancing gender equity and improving health outcomes globally.

Many West African countries report alarmingly high rates of GBV [2], as evidenced by National Demographic and Health Surveys (DHS) in countries like Nigeria, Ghana, and Sierra Leone [3, 10–12]. In Mali, for instance, up to 80% of women experience violence, often linked to patriarchal practices, harmful gender norms, and conflict [2]. Nigeria DHS, reveals that approximately 33% of women have experienced physical or sexual violence, with many subjected to abuse from an early age [3]. The prevalence of physical violence among Nigeria women fluctuated from 11% in 2013 to 14% in 2018 [3, 13], reflecting the complex interplay of societal changes, policy intervention, and persistent gaps in enforcement. A broader regional view shows similarly troubling patterns. In Sierra Leone, 61% of women aged 15–49 have experienced emotional, physical, or sexual violence perpetuated by their intimate partners, which is higher than the 56% rate reported in 2013 [12]. Ghana’s 2024 DHS indicates that 36% of women reported experiencing emotional, physical, or sexual violence, with 33% of women and girls aged 15–49 reporting physical violence, while 25.5% reported psychological violence [10]. Liberia’s 2019–20 DHS found that 60% of women had experienced physical violence, and 9% sexual violence [14]. In Burkina Faso, the 2021 DHS recorded 29% of ever-partnered women and girls have experienced some form of violence, with 20% of women experiencing physical violence and 15% sexual violence [11]. These figures underscore the varying and widespread nature of GBV across West Africa and highlight the urgent need for coordinated, evidence-based interventions.

The impact of GBV extends beyond immediate physical injuries to long-term psychological trauma and socio-economic consequences [6, 15, 16]. Victims often suffer from depression, anxiety, and post-traumatic stress disorder (PTSD), which can hinder their ability to participate fully in society and the economy [6, 15, 16]. GBV can incur significant economic costs, such as healthcare expenses, legal fees, and decreased productivity. These factors contribute to a cycle of poverty and inequality, particularly affecting women and girls in low-income and humanitarian settings [2, 6].

In conflict zones, such as Burkina Faso and Mali, GBV is used as a weapon of war, including sexual violence and exploitation [2, 17]. Similarly, the Boko Haram insurgency in Nigeria has been notorious for using gender-based violence as a weapon of war [18–20]. This terrorist group systematically target both males and females, subjecting them to rape and sexual slavery [18–21]. Comparable dynamics have been observed in post-conflict settings like Sierra Leone and Liberia. This issue is often compounded by significant structural neglect and lack of protection for victims of disasters and insurgency, poor reporting mechanisms, and limited survivor support services [2, 17]. These intersecting vulnerabilities highlight the need for comprehensive, multi-level interventions that address both systemic barriers and individual agency in the prevention and response to GBV in conflict and post-conflict environments.

Efforts to combat GBV have been multifaceted, involving legal reforms, capacity building of health providers, civil society organizations, and support services for survivors [5, 22, 23]. However, challenges remain in the implementation of interventions, enforcement of laws, and changing deeply entrenched cultural norms [1, 24]. In many regions, including West Africa, GBV survivors face barriers to accessing justice and support services [1, 2, 24]. Inadequate policy implementation exacerbates the situation, as laws and guidelines designed to protect survivors are not consistently enforced [1, 24–26].

Despite the publication of several reviews, many of them fall outside the scope of this study [27–30]. These reviews tend to focus on particular intervention settings, such as those in healthcare settings, or they encompass articles from various developed countries, Africa, and the sub-Saharan Africa region [27–30]. While there are studies addressing GBV interventions in West Africa [31–33], the region lacks a comprehensive synthesis of these efforts to underscore lessons about what can and cannot work. We have only seen such synthesis in Eastern and Southern Africa [34, 35]. Synthesizing evidence is essential for pinpointing similarities and gaps to guide future research, especially in West Africa, which faces distinct challenges and features related to GBV. There is an urgent need for focused research to better understand regional nuances and identify specific interventions suitable for West African countries, thereby revealing gaps in current strategies.

Given the complexity and breadth of GBV across West Africa, including studies, a scoping review was considered relevant to begin pulling ideas and findings together in order to effectively map key concepts, identify gaps in the literature, and overall, synthesize evidence from diverse sources. In the context of GBV in West Africa, existing studies vary widely in design, scope, and quality, encompassing qualitative methods, project reports, and quantitative surveys. A scoping review allowed for the inclusion of this broader range of evidence. This approach aligns with the methodological guidance provided by Arksey and O’Malley [36], which recommends scoping reviews for synthesizing emerging or fragmented studies.

Hence, our study aims to synthesize existing evidence on interventions addressing gender-based violence (GBV) in West Africa. The review will map existing health interventions, the varying implementation mechanisms, and the outcomes measured by these interventions. It will also highlight the roles of various stakeholders in GBV intervention. The study findings can inform future policy and guide program designs that can optimize intervention strategies for GBV survivors in West Africa.

## Main text

### Protocol registration

The methodological framework proposed by Arksey and O’Malley [37] guided the structure of this study. Moreover, the checklist from the Preferred Reporting Items for Systematic Reviews and Meta-Analyses (PRISMA) extension tailored for scoping reviews guided the presentation of our study [38]. The protocol for this scoping review was published in the Open Science Framework (OSF) in December 2024 [39].

### Search strategy

A comprehensive literature search was conducted from December 2024 to March 2025 utilizing five key databases: MEDLINE, Google Scholar, Zendy, ResearchGate, and PubMed. The search for MEDLINE articles was conducted via the Ovid interface. The selected databases are integral to biomedical and health research, providing a substantial repository of peer-reviewed articles and grey literature, such as Google Scholar. The databases feature robust search capabilities, user-friendly interfaces, and advanced filtering options, which enhance the precision and comprehensiveness of the searches. By utilizing these databases, the study is assured of capturing high-quality and pertinent literature on GBV. By operationalizing our three concepts (see Supplementary File 1), we developed two primary Boolean search strings that incorporated truncation and MeSH terms, which were applied across all selected databases. We applied filters to limit the publication timeframe to studies published between 2010 and 2024.

In addition, we expanded our search by exploring various websites, including the United Nations Population Fund (UNFPA), World Health Organization (WHO), Pathfinder International, and United Nations Children’s Fund (UNICEF). These websites were searched to identify relevant project evaluation and technical reports, blog posts, and policy briefs. These were identified using site-specific search tools and manual browsing of thematic sections such as “Gender,” “Health,” “Gender-based violence,” “Female Genital Mutilation”. Manual screening of identified reports involved an initial review of titles and executive summaries to assess relevance to GBV interventions in West Africa. Quality assessment was based on source, date of publication, and alignment with the study’s inclusion criteria. All reports, blog posts, and published briefs that did not clearly present intervention mechanisms and outcomes were excluded. Additionally, documents lacking a clear methodology or relevance were excluded.

The review encompassed observational studies and experimental designs, including randomized controlled trials, non-randomized control trials, and systematic reviews that adhered to the established inclusion criteria. This scoping review incorporated project reports and studies of various research methodologies, including qualitative, quantitative, mixed methods, and action/participatory research. Two primary search strings were developed and employed across all databases based on three concepts: (i) gender-based violence, (ii) interventions, and (iii) West Africa. The classification of the West African countries for this study was based on the accepted regional grouping defined by the United Nations geoscheme^1^ for Africa to ensure consistency and alignment with previous regional reviews.

The specific scoping review concepts and search terms are detailed in the supplementary file one. An Excel spreadsheet was meticulously designed to facilitate the compilation of article citations. This spreadsheet includes the following headers: (i) document source (e.g., PubMed or Google Scholar), (ii) search term, (iii) complete reference or citation of the article, (iv) website link, (v) availability of the full text or abstract, (vi) region or country, and (vii) type of document (i.e., primary research, systematic review, or report).

### Eligibility criteria

In order to capture a comprehensive and up-to-date range of interventions, this review exclusively considers studies published from January 2010 to December 2024. The inclusion criteria include: (i) a reported intervention must be present, (ii) the interventions must address specific forms of GBV, including sexual violence and abuse, rape, intimate partner violence (encompassing physical, sexual, and emotional aspects), domestic violence, and female genital mutilation. Only articles or reports published in English are eligible for inclusion in this study. The focus is specifically on articles and reports regarding interventions implemented in West African nations. To be considered for inclusion, interventions must primarily target adults (men and women) and adolescents (boys and girls) aged 10 years and above, as these groups are mostly directly exposed to GBV-related programs. Conversely, studies that primarily focus on children under the age of 10 were excluded from this review because interventions targeting this age group typically involve parental or institutional mediation and usually differ significantly in design, delivery, and intended outcomes. A detailed description of the inclusion and exclusion criteria is provided in the supplementary file 1.

### Study screening

**Fig. 1:**
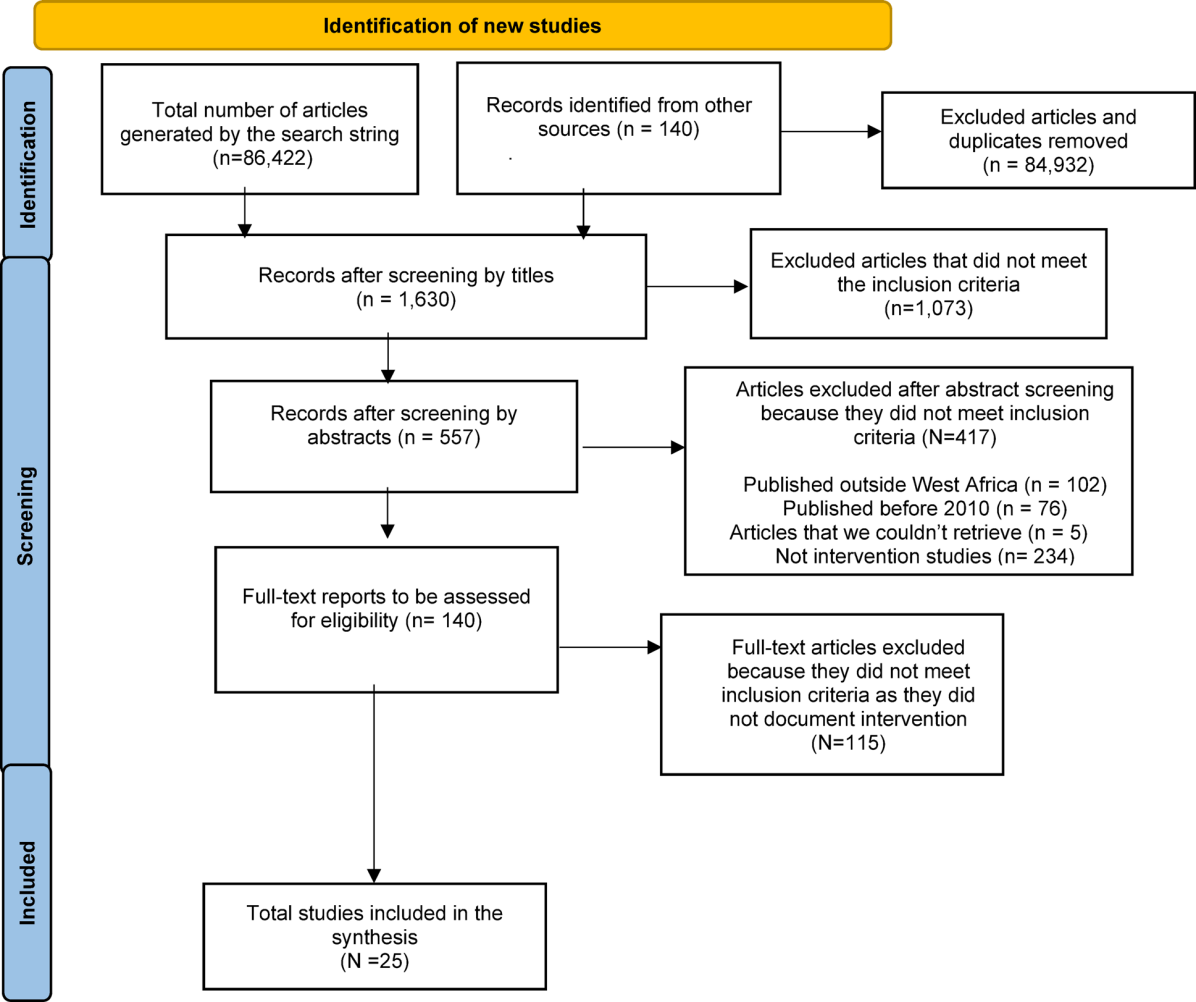
PRISMA Flowchart of the search process

Two independent reviewers conducted a manual screening of titles, abstracts, and full texts. Articles were included based on mutual agreement by both reviewers according to the pre-defined inclusion criteria. In instances of unresolved disagreement, the reviewers convened and invited a third reviewer to jointly assess the article and reached a consensus before proceeding. The article screening process, encompassing both inclusion and exclusion stages, is illustrated in Fig. 1, the PRISMA flow chart. The initial search yielded a total of 86,422 articles across the databases, which were exported to Rayyan [40] for screening. Duplicates were removed prior to applying the eligibility criteria. Subsequently, all records were imported into Zetero for an additional round of duplicate screening through manual verification, ensuring that no duplicates were included in the title and abstract screening phase. After the researchers screened for the titles and removed duplicates, it resulted in 1,630. The reviewers then assessed the abstracts and deemed 557 articles eligible for full-text screening. A thorough examination of the full texts led to the exclusion of 232 articles that did not meet the study’s inclusion criteria, ultimately resulting in the inclusion of 25 studies for analysis.

### Data extraction

Following the compilation of the articles, data were extracted from each article that met the predetermined review criteria utilizing a structured Microsoft Word Excel spreadsheet. The data extraction template included the following columns for documentation: (i) complete reference or citation of the article; (ii) methodology (quantitative, qualitative, mixed methods, review, report, etc.); (iii) country or countries involved; (iv) forms of GBV targeted; (v) type of intervention (e.g., facility-based, community-based, etc.); (vi) description of the intervention (operational definitions); (vii) implementation mechanisms, comprising (a) context, (b) targeted population, and (c) implemented strategy or strategies; viii) actors, stakeholders, and organizations along with their respective roles; and ix) outcomes of the intervention measured.

To elucidate the various forms of GBV interventions, the extracted data were categorized into four typologies: community-based, facility-based, school-based, and media-technology-based interventions. For enhanced clarity, each typology presented data corresponding to the specific forms of GBV that the intervention aimed to address, namely physical violence, sexual violence, or multiple forms of GBV. Furthermore, to describe the diverse mechanisms utilized for delivering these interventions, results were organized according to typology: community-based interventions, facility-based, school-based, and media-technology-driven interventions. Within each typology, the target groups (e.g., men, healthcare providers, women, young individuals, adolescents, etc.), the geographical context (urban, rural, peri-urban) of each intervention were delineated and intervention delivery mechanisms (training/workshops, use of IEC materials, group discussion, text-based strategy etc.). This approach facilitates comparisons across the different intervention typologies.

### Data collation and summarizing of results

This study synthesised the intervention outcomes identified in the review, using the RESPECT Women framework developed by the WHO [41] as a guiding structure. This framework offers a comprehensive approach to evaluating intervention outcomes, encompassing seven key domains: strengthening relationship skills, empowering women, ensuring access to essential services, reducing poverty, creating safe environments, preventing child and adolescent abuse, and transforming societal attitudes, beliefs, and norms [41]. The roles of different stakeholders and organizations involved in GBV interventions were also identified through this review and presented in the result section as one of the major themes.

## Results

A total of 24 peer-reviewed journal articles [31–33, 42–62] and a project report [9], met the inclusion criteria for this review, offering valuable insights into the various response forms to address GBV. Ten of the studies were implemented in Ghana [43, 45–47, 50, 55, 57, 59, 62], six including the project report were in Nigeria [9, 33, 44, 49, 51, 56, 60], and eight articles in other countries including Niger [52, 61], Senegal [31], Guinea [53], Sierra Leone [58], Cote d’Ivoire [32, 48], and Liberia [54]. The result section is structured in three major headings: a typology of existing interventions addressing GBV in West Africa, evidence on the delivery mechanisms of these GBV interventions, evidence on the measured outcomes of these GBV interventions, and different stakeholders involved in addressing GBV in West Africa. The dataset is presented below and in table format in supplementary file two.

### Typology and delivery mechanisms of the GBV interventions

**Fig. 2:**
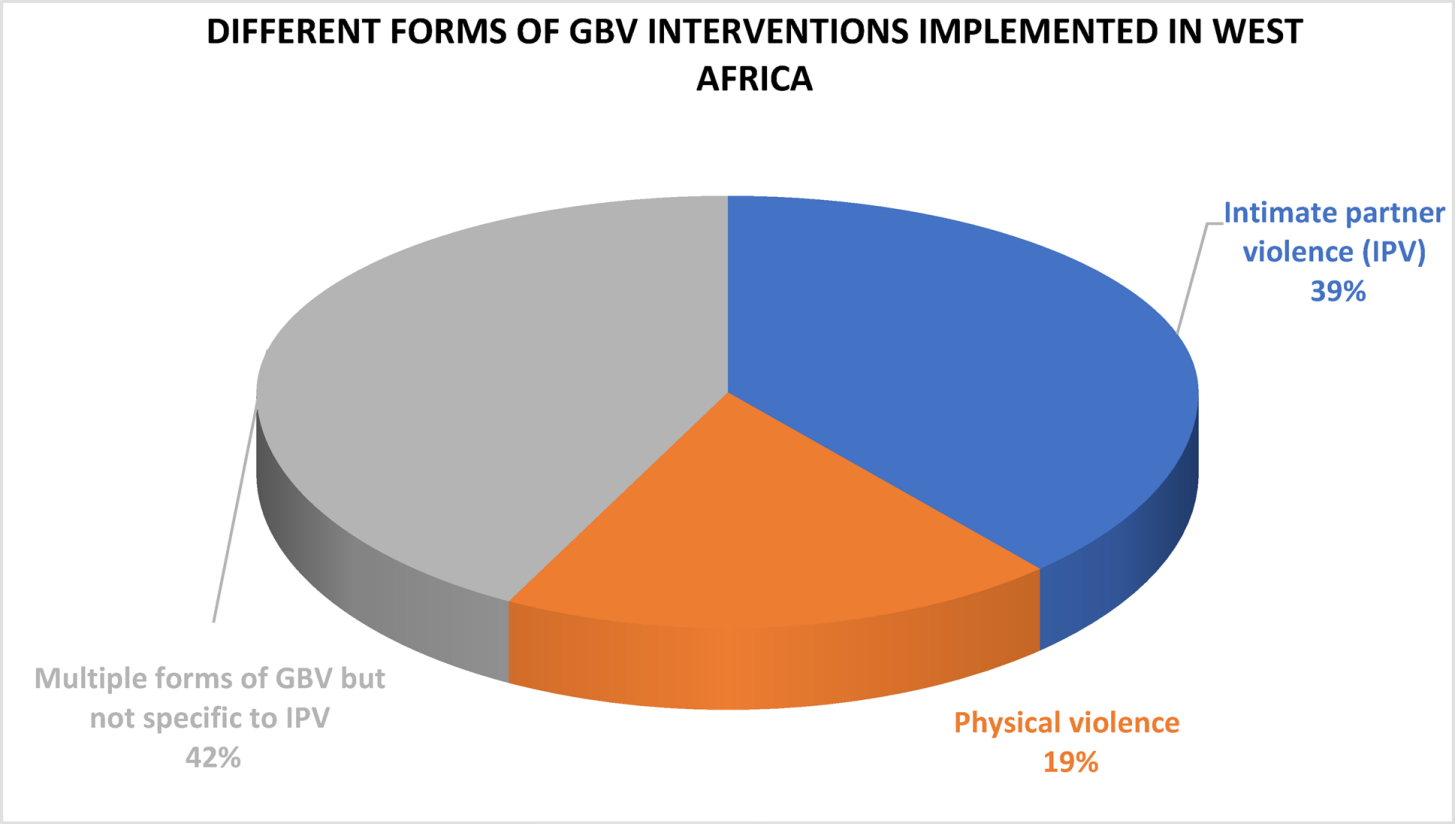
Different forms of gender-based violence intervention implemented in West Africa

The data from the findings are presented systematically to provide a comprehensive understanding of the approaches applied within different settings. About 42% (11) of the articles addressed multiple forms of GBV, 39% (10 articles) specifically implemented interventions to address IPV and a few 19% (5) of these articles reported interventions that addressed physical violence particularly, domestic violence and FGM/C as presented in Fig. 2. Two articles reported multicomponent interventions, a combination of community-facility-based and community-school-based intervention strategies [9, 58].

**Fig. 3:**
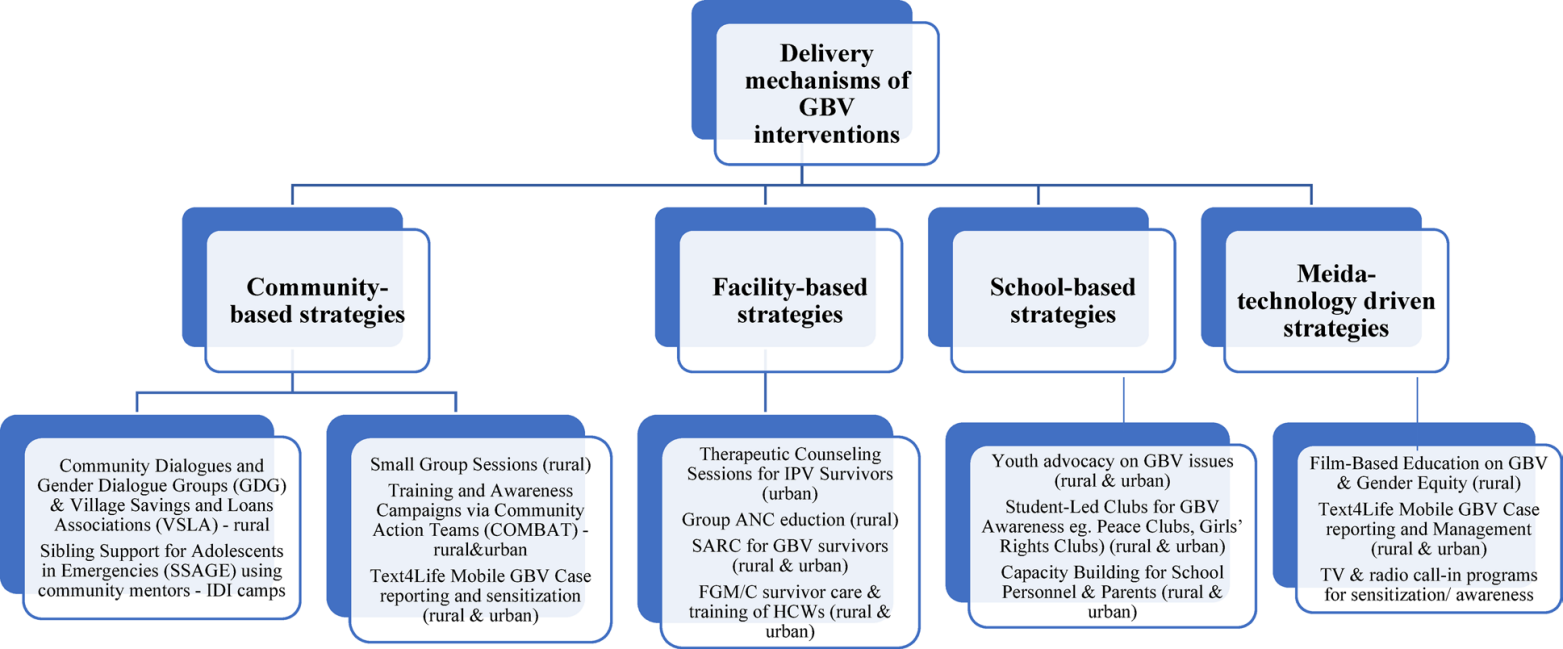
Evidence on the delivery mechanisms of GBV interventions implemented in West Africa

To explore the spectrum of gender-based violence (GBV) interventions implemented across West Africa, this section organizes the findings based on intervention typology. The typology is community-based interventions, facility-based interventions, school-based interventions, and media-technology-based interventions. The delivery mechanisms of these GBV interventions are also presented in Fig. 3.

The review also identified interventions that targeted primary, secondary, and tertiary GBV prevention. In this study, the primary prevention is defined as interventions that aim to stop gender-based violence before it occurs by addressing root causes such as harmful gender norms, promoting respectful relationships, and fostering gender equality through awareness, education, and community engagement. Secondary prevention is defined as an early intervention after violence has occurred, providing immediate support to survivors, such as crisis counselling, medical care, and legal protection, to prevent further harm and escalation. While the tertiary preventions are interventions that seek to reduce the long-term impact of GBV by offering rehabilitation for perpetrators and trauma-informed care for survivors, helping them recover and reintegrate into society.

### Community-based interventions

A total of twenty-one articles documented interventions implemented at the community level. Of these, four interventions specifically addressed physical violence [50–53], while seventeen targeted multiple forms of GBV within their scope [9, 31, 32, 43, 45–49, 55–62]. Notably, none of the reported interventions focused exclusively on emotional or psychological violence.

#### Physical violence

Our scoping review identified four scholarly articles that examined community-based interventions addressing physical violence in Ghana, Nigeria, Guinea, and Niger [50–53]. The interventions varied in scope, with those in Nigeria and Niger focusing primarily on the prevention of physical and domestic violence against adolescent girls within internally displaced persons (IDP) camps [51, 52]. The two studies incorporated mentorship and dialogue sessions where respected community members facilitated weekly discussions with adolescent girls, their male siblings, and caregivers [51, 52].

In contrast, the intervention in Ghana targeted secondary prevention efforts, specifically addressing the needs of women who are survivors of domestic violence [50]. The Guinea community-based intervention component had a focus on primary prevention of FGM/C by creating awareness and advocacy visits at the community level [53].

In Nigeria, Koris et al. explored the implementation of gender-transformative, whole-family support programming aimed at preventing physical violence against adolescent girls in Northeast Nigeria [51]. Their study highlighted both opportunities and challenges inherent in this approach. Similarly, a study investigated the innovative use of the brother-sister sibling dyad as a mechanism for preventing gender-based violence [52]. This intervention engaged male siblings in family-strengthening programs within humanitarian contexts in Niger, with a specific focus on reducing physical violence against adolescent girls perpetrated by male siblings and caregivers [52].

In Ghana, researchers provided a nuanced evaluation of secondary prevention efforts targeting domestic violence survivors [50]. Their study examined the experiences of Ghanaian women in accessing formal support services, drawing on provisions within the Ghana Domestic Violence Act and expert insights to assess the effectiveness of these measures [50].

#### Multiple forms of GBV

We identified a total of seventeen articles that documented community-based interventions aimed at addressing multiple forms of GBV across various West African countries [9, 31, 32, 43, 45–49, 55–62]. These interventions encompass both primary and secondary prevention efforts and address diverse manifestations of GBV, including violence against women and girls, intimate partner violence, sexual violence, physical violence, and emotional abuse.

In Ghana, eight articles were identified, of which seven described interventions focusing on the primary prevention of GBV, while one detailed a secondary prevention approach [43, 45–47, 57, 59, 62]. Among these, two documented interventions were implemented across multiple African countries, including Ghana [46, 62]. Four articles specifically targeted the prevention of violence against women and girls at the community level [43, 46, 47, 62], while three targeted intimate partner violence, encompassing emotional, physical, and sexual violence perpetrated by intimate partners [45, 55, 57]. Furthermore, one intervention combined primary and secondary prevention to comprehensively address all forms of GBV in Ghana [59]. The community-based initiative aimed at preventing IPV among women in predominantly rural areas used unconditional cash transfers and health insurance premium waivers, providing financial relief to pregnant and lactating mothers in low-resource households [45]. Similarly, the Community Action Team (COMBAT) intervention was implemented in rural, peri-urban, and urban districts of Ghana to prevent violence against women and girls [43, 46, 47, 55, 62]. COMBAT members conducted awareness campaigns in churches, at festivals, and via radio programs while facilitating referral services and follow-up interventions for GBV survivors [43, 46, 47, 55, 62].

Four articles reported interventions implemented in Nigeria [9, 49, 56, 60]. Two focused on primary prevention measures aimed at reducing violence against women [49, 60], while two others highlighted secondary prevention strategies that involve the establishment of reporting mechanisms and the provision of post-GBV services to victims of sexual, emotional, and physical violence [9, 56]. The sexual gender-based violence (SGBV) prevention study was implemented in both rural and peri-urban areas, using small group discussions and community dialogues to reflect on gender equity, SGBV, and increase family planning uptake among young couples [60].

Five articles documented interventions addressing GBV in Niger, Senegal, Sierra Leone, and Côte d’Ivoire [31, 32, 48, 58, 61]. Two articles described interventions conducted in Côte d’Ivoire, with one focusing on IPV involving physical and sexual violence [32]. This study combined financial empowerment with structured dialogue sessions through the Village Savings and Loans Associations (VSLA) model, addressing gender inequities in households and reducing IPV among women and their male partners [32]. The other study carried out in Côte d’Ivoire focused on engaging men to reduce the incidence of GBV achieved through structured discussion groups that encouraged behavioural change and accountability within relationships [48]. The male-focused discussion groups helped shift harmful gender norms and encouraged IPV reduction [48], while Gender Dialogue Groups (GDG) combined financial literacy with structured household discussions to reinforce equitable decision-making [32].

In Niger, Silverman and colleagues examined an intervention aimed at addressing intimate partner violence among adolescent mothers [61]. Meanwhile, in Sierra Leone, Reilly reported on community-level efforts to reduce sexual violence, physical violence, and abuse among adolescent girls [58]. Regional initiatives across 120 villages in two regions of Senegal were documented in an article by [31], showcasing intervention focused on addressing GBV among adolescent girls and young women at the community level.

### Facility-based interventions

 A total of 12 articles were found to report facility-based interventions to address either physical violence or multiple forms of GBV [9, 33, 42–44, 46, 47, 53, 54, 56, 59, 62]. These interventions were implemented across rural, urban, and peri-urban settings, strengthening services for survivors and improving healthcare worker capacity.

#### Physical violence

This scoping literature review identified two articles reporting facility-level secondary prevention interventions targeting physical forms of GBV, specifically FGM/C [53, 54]. These studies provided insights into innovative approaches designed to enhance case management and communication in healthcare settings across Liberia and Guinea. The study in Liberia focused on case identification and management of FGM/C survivors through the use of a novel mobile application introduced in both urban and rural regions [54]. This intervention took a significant step forward in building capacity of healthcare workers with the knowledge and skills required for effective and ethical management of FGM/C cases. This was achieved through training workshops and use of mobile learning apps.

An intervention in Guinea and two other non-West African countries emphasized the need for person-centred communication (PCC) as a strategy for FGM/C prevention [53]. This approach aimed to address the medicalization of FGM/C by targeting health workers actively involved in the practice. The healthcare workers were trained to create awareness of FGM/C during ANC sessions. They actively engaged ANC clients through counselling services by applying a person-centred care and communication approach [53].

While the study in Liberia emphasized technological solutions for self-assessment and skill-building [54], the intervention in Guinea highlighted the critical role of communication in shifting practices and attitudes related to FGM/C [53].

#### Multiple forms of GBV

In addressing more than one form of GBV, we identified nine articles that reported secondary prevention efforts in both Nigeria [9, 33, 44] and Ghana [43, 46, 47, 55, 59, 62]. These studies implemented interventions that employed secondary prevention strategies to address existing cases of IPV and sexual and gender-based violence (SGBV). One of the studies implemented in Ghana prioritized proactive primary prevention measures to mitigate the risk of IPV before it occurred [42]. The study specially implemented a group antenatal care (ANC) intervention in low-resource settings (rural areas) focused on primary prevention of IPV among pregnant women through education sessions on healthy relationships [42]. This approach incorporated storytelling, peer support, and skill-building exercises to reinforce emotional resilience.

Two studies from Nigeria primarily emphasized secondary prevention mechanisms aimed at addressing existing IPV cases and mitigating its impacts [33, 44]. Akor et al. conducted a single-blinded randomized controlled trial among pregnant IPV victims, using the Abuse Assessment Scale to recruit seventy-two participants who were evenly divided into intervention and control arms [44]. Their study applied a quantitative research approach, demonstrating its potential to reduce IPV during pregnancy. The intervention used therapeutic counselling strategy for pregnant women experiencing violence, guiding them through biweekly problem-solving therapy sessions.

Bamigboye and colleagues implemented a customized educational training program for midwives to improve their capacity to detect and manage IPV cases effectively [33]. Similarly, another study targeted a broader spectrum of SGBV by strengthening responses through ten existing Sexual Assault Referral Centers (SARCs) across nine states in Nigeria [9]. The intervention focused on improving the centers’ capacity to deliver sustainable and comprehensive services for survivors while fostering collaboration with governments, communities, and organizations. This study was implemented across Nigeria, offering primary prevention effort through sensitization campaigns, secondary prevention effort through forensic medical services and trauma counselling, and tertiary prevention through referrals for legal services and skills acquisition programs [9]. Most of the SARCs were situated in tertiary and secondary healthcare facilities, while two SARCs were free-standing independent centres. However, the report showed that none of the SARCs were situated at the primary healthcare facilities that serve the grassroots. These SARCs ensured survivors had access to medical and psychological support [9].

In Ghana, the intervention reported by Abdelnabi et al. was centred on primary prevention strategies designed to proactively address IPV [42]. The implementers introduced a facility-based intervention where women maintained continuous engagement with the same midwife throughout their pregnancy [42]. This approach incorporated innovative techniques such as storytelling, picture cards, role-play, and demonstrations tailored to each gestational stage for effective empowerment of pregnant women with knowledge and skills to mitigate the risk of IPV during pregnancy. Across Ghana, a national-level intervention combined both facility and community based strategies as the project integrated free treatment services, counselling support, prevention campaigns in public spaces such as churches, markets, homes and school [59]. The project also established referral networks, ensuring survivors had access to specialized medical and psychological assistance [59].

### School-based intervention

The review identified only two articles detailing a school-based intervention aimed at addressing gender-based violence (GBV) in Sierra Leone [58] and Nigeria [9].

#### Multiple forms of GBV

The study focused on the experiences of adolescent girls subjected to violence in educational settings [58]. This primary prevention initiative targeted school-related gender-based violence (SRGBV) by working to reduce instances of physical violence, sexual violence, and abuse in schools. Additionally, the intervention sought to increase the reporting of such cases, fostering a safer and more supportive school environment for adolescent girls.

Efforts to prevent school-related GBV (SRGBV) were implemented in both urban and rural communities, focusing on awareness and student engagement [9, 58]. In Sierra Leone, interventions aimed to reduce physical and sexual violence in junior secondary schools by integrating teacher training, student clubs, and capacity-building programs for parents [58]. The capacity-building sessions equipped teachers and parents to address violence in schools, while the peace clubs, girls’ rights clubs, and literacy debates fostered student engagement [58]. Nigeria’s SARC outreach campaign extended prevention efforts into educational spaces, training school personnel while promoting GBV awareness among students [9]. These studies did not specify whether the intervention schools were public or private [9, 58], but the Nigeria SARC program highlighted the involvement of both formal and informal educational settings [9]. The informal education setting involved the establishment of centres within the communities, called second chance education centre, to improve the literacy level of women and girls who were not opportune to attend and complete their formal education.

### Media and technology-based interventions

Digital and media-driven strategies played a significant role in GBV prevention. The review identified three articles implemented in Senegal [31] and Nigeria [9, 56] that utilized medial based strategies in addressing to GBV issues.

#### Multiple forms of GBV

In Senegal, film-based education programs utilized biweekly screenings and post-viewing discussions to shift attitudes among adolescent girls and young women, covering topics such as IPV, workplace violence, forced/child marriage, rape, and gender equity [31]. Complementary workshops guided reflections on personal and community-level change, with trained facilitators ensuring sustained engagement. The Text4Life initiative in Nigeria leveraged mobile phones to streamline GBV reporting channels and enhance service accessibility [56]. The intervention also involved community sensitization, and facility-based response mechanisms. Awareness campaigns through radio, television, town hall meetings, and social media strengthened outreach efforts, ensuring survivors could access necessary support [56]. Another study implemented in Nigeria also reported the awareness creation through radio and television (TV) phone-in programs [9].

## Evidence on the measured outcomes of these GBV interventions

The findings of this review will be guided by the RESPECT Women framework, which has been adapted to analyse the measured outcomes across various intervention typologies. The measured outcomes will be presented using the seven elements of the RESPECT Women Framework [41], which include: i) Strengthened relationship skills, ii)Transformation of attitudes, beliefs, and norms, iii) Creation of safe environments, iv) Prevention of adolescent abuse, v) Enhanced and ensured access to essential services, vi) Reduction of poverty, and vii) Empowerment of women. In presenting the outcomes, the domain for empowerment of women and the reduction of poverty is discussed as on sub-theme. It is notable that some interventions assessed more than one outcome, and these will be presented according to the relevant element.

### Strengthened relationship skills

This scoping review identified several interventions that measured outcomes aligning with the RESPECT Women framework element on “Relationship skills strengthened”. Some articles reported that the interventions addressed foundational relationship dynamics among couples and promoted long-lasting positive change by fostering effective communication, conflict management, and mutual understanding.

Specifically, in Nigeria a project had significant success in reducing physical intimate partner violence (IPV) within intervention arms compared to the control arm [49]. While trends suggested declines in emotional IPV across all intervention arms, these effects were reported to be only marginally significant in specific cases, and no significant reductions were observed in sexual IPV. The intervention notably did not focus directly on IPV issues; instead, it aimed to shift couple relationship dynamics by addressing traditional gender norms and offering relationship education and skills-building. These efforts successfully improved communication, conflict management, and mutual respect among couples, contributing to sustained reductions in physical IPV over time. However, the study did not explicitly clarify whether self-reporting of violence incidents increased or decreased as a result of the intervention, despite reporting reductions triggered by survey questions.

Similarly, Shaw and colleagues conducted an intervention in Nigeria targeting equitable and violence-free relationships among young couples, supported by faith leaders [60]. This program achieved notable reductions over time in reports of emotional, physical, and sexual IPV among women and certain religious subpopulations. Women, in particular, exhibited substantial declines in reported experiences of emotional IPV, unlike men. Additionally, the intervention improved relationship quality and conflict resolution among participants. Both men and women displayed significant intentions to adopt nonviolent conflict resolution methods as an alternative to IPV. The intervention also improved participants’ perception of relationship quality over time, indicating its success in fostering healthier relational dynamics. Another Nigeria article that aimed to address violence against adolescent girls in their various households, reported increased communication among family members of adolescent girls [51]. Enhanced communication emerged as a critical pathway for violence prevention, as adolescent girls expressed greater comfort in sharing their thoughts and emotions with their fathers. Similarly, Fathers’ improved communication styles encouraged broader reflection among family members, fostering more equitable interactions within households.

One of the Ghana studies reported how relationship-focused pathways strengthened intimate relationship skills and quality among participants [62]. The intervention emphasized constructive communication and conflict resolution, which helped mitigate and prevent violence. Female community members reported feeling more equipped to manage anger and impatience through the program, gaining a better understanding of how specific behaviours, such as anger, could contribute to IPV. This awareness and knowledge translated into improved relational dynamics and enhanced participants’ capacity to foster violence-free relationships.

### Transformed attitudes, beliefs, and norms

The findings from the scoping review highlight transformative shifts in both individual and community attitudes toward gender-based violence (GBV) and gender equity resolution [31, 32, 45, 51, 60, 62]. Across multiple interventions, participants reported a deeper understanding of intimate partner violence (IPV), including the recognition of coerced sex as a form of violence. In Nigeria, intervention programs successfully fostered gender equity by encouraging male engagement in household roles and promoting non-violent conflict resolution [51, 60]. These initiatives also strengthened community cohesion and interfaith dialogue, suggesting a broader cultural shift toward equitable relationships. Similarly, efforts to reshape sibling dynamics in Nigeria and Niger showed positive results, with male siblings demonstrating greater emotional involvement and support for their sisters’ safety [51, 52]. However, the persistence of patriarchal norms such as restricting female movement in the name of protection, underscored ongoing challenges in dismantling entrenched gender hierarchies.

In Ghana, community-level interventions led to a re-evaluation of traditional gender roles, fostering gender-equitable decision-making within households and promoting collective dialogue. Men in intervention communities began engaging more in domestic responsibilities, demonstrating gradual shifts in family structures [45, 62]. Additionally, while economic interventions such as cash transfers improved financial stability and reduced household conflict, they were less effective in fundamentally altering gender norms in contexts of economic insecurity. Across all studies [32, 45, 62], IPV exposure declined to varying degrees, with reductions in economic abuse and acceptance of wife beating particularly evident among high-adherence groups participating in structured discussions and savings programs [45]. Male engagement played a crucial role in these shifts, with intervention groups reporting greater involvement in household duties and improved conflict management strategies. While these findings suggest meaningful progress, they also highlight the need for sustained, multi-faceted approaches to challenge deeply rooted gender inequalities and promote lasting change.

Similarly, in Senegal, the television-based edutainment program successfully engaged female audiences, with high participation rates and significant responsiveness to its messaging on gender-based violence (GBV), sexual and reproductive health (SRH), and maternal and child health (MCH) [31]. The intervention fostered social dialogue, encouraged behavior change, and demonstrated the potential of media-driven awareness campaigns in shifting harmful norms limiting access to services. Despite variability in attendance across regions, the approach proved effective in initiating conversations on sensitive topics, though additional educational components may be necessary for sustained impact.

### Prevention of violence and abuse against adolescents

The review identified three studies that focused on interventions designed to prevent violence and abuse against adolescents, particularly in the context of familial dynamics and sibling relationships [31, 51, 52]. The study conducted in Niger demonstrated meaningful reductions in the perpetration of physical violence by male siblings within participating families [52]. A key outcome of the intervention was the reconceptualization of male siblings’ roles in ensuring their sisters’ safety, with boys developing more protective and supportive behaviors toward their adolescent sisters. This shift reflects a broader redefinition of masculinity within family structures, emphasizing non-violent approaches to safeguarding female relatives. Similarly, in Nigeria, the intervention produced comparable positive effects. Participants reported a strengthened understanding of gendered power dynamics and significant reductions in violence perpetration across various household interactions [51]. The intervention facilitated deeper discussions on gender roles, encouraging male siblings to adopt more equitable perspectives and reduce harmful behaviors within the home. While these studies show promising results in reshaping familial relationships and reducing adolescent-targeted violence, they also highlight the continued need for sustained engagement to challenge entrenched gender norms that contribute to violence within households.

### Creating safe environments

We found several articles that reported measured intervention outcomes that fit under the “Environments Made Safe” element of the RESPECT Women framework campaigns [31, 43, 55, 56, 58, 62]. Many of the articles that implemented campaign and sensitization programs reported (i) increased awareness of IPVs, (ii) availability and awareness of reporting structures, (iii) availability and awareness of healthcare services for victims, as well as (iv) the implementation of legal protection laws. These articles emphasized that supportive environments were achieved by ensuring the continuous engagement of community members in GBV prevention and response efforts. Sensitization efforts helped communities recognize and address the structural barriers preventing survivors from seeking justice and support.

By increasing awareness of the reporting channel through the text4life device, Okonofua et al. reported that their intervention effectively mobilized community participation, enabling individuals to report GBV cases, often on behalf of others [56]. Community-level settlements often received strong support from local leaders. The focus on enhancing reporting mechanisms and raising awareness contributes to fostering supportive structures, ensuring survivors can access the help they need and encouraging collective accountability within the community. Additionally, the intervention improved access to services and follow-up mechanisms, with many participants reporting that the channel made it easier to report repeated abuses and seek help. However, challenges remained, as some participants highlighted inconsistent responses from service providers and hesitancy to pursue legal action against perpetrators.

Similarly, in Ghana, community-led programs such as COMBAT played a crucial role in household sensitization through home visits and public awareness campaigns [43, 55, 62]. Stern and colleagues further emphasised on the role of enabling environments at the community level, where the multi-component intervention supported leadership engagement and connected families to vital support systems [62]. For instance, the program successfully curbed harmful practices such as property dispossession of widows, demonstrating its role in addressing broader community issues alongside IPV. Meanwhile, in Sierra Leone, school-based violence reduction interventions made notable strides in ensuring safer learning environments for girls and boys [58]. Mid-term evaluations showed a decline in flogging and teacher-perpetrated sexual exploitation, fostering a more protective atmosphere. The reduction in sex-for-grades transactions and increased reporting of abuse suggest growing confidence among students to challenge harmful practices. However, lingering barriers such as fear of retaliation and societal stigma underscore the continued need for sustained interventions. Awareness-raising initiatives around teacher codes of conduct and gender rights proved instrumental in driving social change, encouraging more individuals to speak up against abuse and advocate for safer environments. Strengthening education and fostering accountability at all levels remain pivotal in ensuring survivors receive the necessary protection and support.

### Access to services enhanced and ensured

The findings from various community-based interventions across Ghana, Niger, and Côte d’Ivoire reveal both progress and persistent challenges in ensuring services for women affected by GBV and IPV [9, 32, 48, 50, 57, 59, 61, 62]. Overall, intervention efforts showed promising trends in reducing IPV, improving access to support services, and increasing contraceptive use, improving the knowledge, capacity and skills of the healthcare service providers in case identification, management, documentation and referrals [9, 32, 33, 42, 48, 50, 57, 61, 62]. Efforts to strengthen SARCs and establish direct links between survivors and supportive services contributed to enhanced accessibility, but additional efforts are needed to ensure sustainability [9]. While some programs demonstrated measurable impact, gaps remained in institutional support, survivor protection, and knowledge dissemination. Notably, in Ghana, barriers such as lack of trust in formal support channels, limited awareness of legal resources like support centres for victims of violence, lack of privacy at the support centres, and fear of partner arrest prevented women from fully utilizing available services [59]. Economic dependence and low formal education further hindered survivors from seeking justice or support. Survivors expected governmental provisions for shelter, medical care, and legal aid, yet many reported experiencing revictimization within formal service structures [50].

In Niger, interventions promoting modern contraceptive use led to substantial increases in uptake, particularly among women engaged during household visits and group-based educational sessions. However, IPV reduction, though evident, lacked statistical significance in certain intervention arms, underscoring the complexity of behaviour change and the need for sustained efforts. Across the studies, women’s exposure to IPV showed varying degrees of decline [32, 48, 50, 57, 59, 61]. However, structural limitations such as inadequate resources, lack of privacy in health service support units for victims of violence, and logistical constraints remained key obstacles in delivering health services. Survivors who had contact with women’s rights organizations showed a higher awareness of legal and psychosocial support options, but many were still unaware of their full entitlements under domestic violence laws [50, 59].

The scoping review also identified facility-based interventions aimed at addressing FGM/C through education and technology [53, 54]. In Liberia, the mLearning app for FGM/C survivor care was found to be an effective tool for healthcare providers, enhancing knowledge, clinical decision-making, and follow-up care [54]. The app facilitated self-learning and broadened awareness among both providers and patients, contributing to improved competence in managing FGM/C-related complications. While many providers viewed themselves as advocates against FGM/C, they emphasized that education alone is insufficient; legal enforcement and engagement with traditional and religious leaders are crucial for meaningful change. A facility-based intervention in ANC clinics further demonstrated the feasibility and acceptability of integrating FGM/C prevention into maternal healthcare settings [53]. Most ANC providers found the person/client-centred communication approach to be a practical strategy for discussing FGM/C prevention, with training reinforcement identified as a crucial element for long-term implementation. Clients reported satisfaction with the discussions, recognizing healthcare workers as appropriate messengers due to their clinical expertise and cultural awareness. The intervention significantly enhanced providers’ ability to deliver FGM/C prevention messaging, while clients exposed to the program exhibited a lower likelihood of supporting the practice. While adherence to distributed resources varied, the intervention reinforced the importance of structured messaging within ANC clinics. Impact assessments highlighted shifts in knowledge, attitudes, and reported intentions among both providers and clients, indicating the potential for healthcare-based interventions to play a significant role in preventing FGM/C.

### Economic empowerment and poverty reduction

The scoping review identified three studies that examined poverty-related issues in relation to gender-based violence [32, 45, 57]. Findings indicated that poverty is a key determinant of IPV, and by reducing financial insecurity, the intervention contributed to lower household conflict, increased empowerment and improved emotional well-being [32, 45]. Participants reported that increased economic security and empowerment helped mitigate IPV risk, suggesting that poverty alleviation initiatives play a crucial role in fostering more supportive and less violent households and communities [45]. Women who gained financial independence experienced a shift in household power structures, allowing for improved decision-making and reduced dependency on abusive partners. While these interventions demonstrated positive outcomes, findings underscored the need for sustained engagement beyond direct financial assistance to ensure lasting reductions in IPV [45, 57]. One of the studies showed that a significant percentage of women were experiencing various forms of IPV, including controlling behaviors, emotional abuse, physical violence, and sexual violence during the baseline assessment [57]. However, the post-evaluation aggregate data showed that the intervention did not produce statistically significant reductions in IPV within the study’s timeframe, but the negative coefficients suggest a trend toward reduced violence [57]. While this trend is not strong enough to be considered definitive, it indicates that with sustained intervention and further assessment, there may be potential for meaningful impact over time. Structural barriers, such as gender norms that limit women’s access to resources and economic opportunities, must also be addressed to maximize the long-term impact of poverty reduction programs on preventing violence.

## Different stakeholders involved in addressing gender-based violence (GBV) in West Africa

The review identified various stakeholders involved in the fight against gender-based violence (GBV) in West Africa which is presented in Table 1. This involves a diverse set of stakeholders across government agencies, non-governmental organizations, research institutions, healthcare providers, and community leaders. Healthcare providers, including midwives and primary healthcare workers, play a crucial role in facilitating discussions and raising awareness on GBV and female genital mutilation (FGM), ensuring survivors receive medical and psychosocial support [33, 43, 46, 47, 53, 56, 62]. Community leaders, including village elders, chiefs, religious leaders, and teachers, contribute to awareness campaigns by fostering conversations within communities and promoting behaviour change. Government entities such as the Ministry of Health (MOH), Ministry of Women Affairs and Social Development (MWASD), and Ministry of Justice (MOJ) oversee policy implementation, legal protection, and service delivery for GBV survivors [9, 45, 50–52]. In collaboration with the Ministry of Education (MOE), these agencies also integrate gender equity education into formal and informal learning spaces.

Several international and national organizations have been instrumental in implementing GBV interventions. Mercy Corps Nigeria (MCN) trained local mentors to facilitate discussions on gender dynamics, while Pathfinder International and the Women’s Health and Action Research Centre (WHARC) collaborated with UNFPA to support GBV projects [51, 52]. Innovations such as Text4Life in Nigeria introduced mobile-based reporting channels, allowing survivors to seek assistance through civil society organizations (CSOs) and primary healthcare centres. Research institutions, including Washington University in St. Louis, Yale School of Public Health, and Innovations for Poverty Action, conducted evaluations to assess program effectiveness. Funding support has been provided by global agencies such as USAID, UNICEF, the World Bank, and the Government of Canada through Global Affairs Canada [9, 45, 50–52]. Programs like the Justice for All initiative and the Managing Conflict in Nigeria Program, backed by the UK’s Department for International Development and the European Union, contributed to the establishment of SARCs. The collective efforts of these stakeholders demonstrate a multi-sectoral approach to GBV prevention, emphasizing legal enforcement, survivor support, education, and community-driven change.

**Table 1:** Identified stakeholders and roles in GBV prevention and support

Category	Stakeholders/organizations	Roles
Government Agencies	Ministry of Health (MOH)	Oversees management of sexual and gender-based violence (SGBV) health issues and directs implementation of interventions (including FGM/C)
	Ministry of Women Affairs and Social Development (MWASD)	Ensures gender equity policies are implemented and provides holistic support for GBV survivors.
	Ministry of Justice (MOJ)	Provides legal support and justice services for GBV survivors.
	Ministry of Education (MOE)	Oversees education and gender awareness programs in both formal and informal settings.
	National Agency for the Prohibition of Trafficking in Persons	Works to combat trafficking-related GBV and ensure survivor protection.
	Department of Social Welfare	Provides social services and victim support programs for survivors of GBV.
	Domestic Violence and Victims Support Unit (DOVVSU)	Enforces domestic violence laws and supports GBV survivors through legal and institutional channels.
	Police force	Police investigation and prosecution
	Commission on Human Rights and Administrative Justice (CHRAJ)	Provides oversight and advocacy for fundamental freedoms while addressing complaints of discrimination and abuse. Responsible for investigating human rights violations, ensuring compliance with constitutional provisions, and offering legal assistance to individuals facing injustice.
Non-governmental organizations	Ark Foundation, International Federation of Women Lawyers (FIDA-Ghana), and Women Alliance	Advocates for women’s rights (collaborators in designing the intervention package)
Implementing Organizations	Mercy Corps Nigeria (MCN)	Trains community mentors and facilitates structured sessions for adolescent girls and caregivers.
	Pathfinder International	Implements GBV-related programs with a focus on reproductive health and community sensitization.
	Women’s Health and Action Research Centre (WHARC)	Implements GBV interventions with UNFPA support, focusing on advocacy and survivor care.
	Education as a Vaccine (EVA)	Conducts awareness campaigns and educational programs on gender issues.
	Planned Parenthood Federation of Nigeria (PPFN)	Provides reproductive health services and supports GBV prevention efforts.
	Civil Society Organizations (CSOs)	Ark Foundation, Women Alliance Ghana, FIDA-Ghana | Advocates for women’s rights and provides legal assistance to GBV survivors.
	Local CSOs supporting the Text4Life initiative	Serve as first responders to GBV cases, ensuring survivors receive medical care and counselling.
Healthcare Providers	Primary healthcare centers (PHCs	Provide medical treatment, counselling, and support for GBV survivors.
	Midwives, nurses, and trained healthcare professionals	Deliver maternal healthcare, discuss FGM/C prevention, and support survivors (including case identification and management).
Community Stakeholders	Village elders, chiefs, religious leaders, and teachers	Initiate discussions on GBV, foster community engagement, and support awareness campaigns.
	Community healthcare volunteers	Assist in local/community implementation of GBV-related interventions and survivor outreach
	Trained GBV champions	Engage communities, educate young adults, and promote gender equality advocacy.
	Child Welfare Committees (CWCs)	At the chiefdom level, the CWCs were established to handle cases of abuse in communities
Research Institutions	Washington University in St. Louis (WashU)	Conducts research and evaluates GBV intervention programs such as SSAGE.
	Yale School of Public Health (YSPH)	Leads studies on gender equity, GBV interventions, and social impact.
	Innovations for Poverty Action (IPA)	Assesses program effectiveness in GBV prevention and community resilience.
	International Rescue Committee (IRC)	Conducts research and provides humanitarian support for GBV survivors
Funding Partners	USAID, UNICEF, UNFPA, World Bank, Global Affairs Canada, the Canadian International Development Agency (CIDA), National Institute for Health	Provide financial and technical support for GBV intervention programs and policy implementation.
	Justice for All program (UK’s DFID)	Funds sexual assault referral centers (SARCs) and legal protection initiatives.
	Managing Conflict in Nigeria Program (European Union)	Supports community-level programs addressing GBV and conflict resolution.

## Discussion

We conducted a scoping review of the literature that documented the typologies, delivery mechanisms, and outcomes of GBV interventions implemented in West Africa. This scoping review reveals a pronounced emphasis on community-based interventions in addressing GBV in West Africa, with the majority of the included studies documenting such approaches. This predominance underscores the critical role of community engagement in both the prevention and response to GBV, particularly in settings where formal institutional support may be limited or inaccessible. Community-based interventions are often lauded for their cultural adaptability, grassroots legitimacy, and potential to shift harmful social norms through participatory strategies [63, 64]. The relatively limited number of studies reporting school-based interventions highlights a critical gap in early prevention strategies. Schools are pivotal environments for shaping gender norms and preventing violence before it begins. The scarcity of school-based programs may reflect challenges in implementation, such as curriculum constraints or lack of institutional support, but it also signals an area for future investment and research.

Our findings indicate that community-based interventions serve as a significant mechanism for addressing GBV, particularly in rural and peri-urban settings where social norms play a dominant role in shaping attitudes toward IPV. Community-driven programs such as Community Action Teams (COMBAT) in Ghana and GDGs in Côte d’Ivoire were effective in fostering discussions on gender norms, IPV, and women’s empowerment. These interventions engaged key community gatekeepers, such as religious leaders, traditional authorities, and local mentors, to create an enabling environment for behaviour change. However, despite their success in raising awareness and promoting attitudinal shifts, challenges remained in sustaining behavioural change beyond the intervention period. Gender norms, particularly those reinforcing male dominance and female subordination, continued to influence relationship dynamics, demonstrating the need for ongoing reinforcement of such programs. A similar community-based approach was implemented in central Africa, engaging males in gender-equity discussions, which showed positive shifts in attitudes toward violence against women [65]. Community-based initiatives represent a valuable form of intervention that can effectively address complex issues such as gender-based violence (GBV), health behaviours, and the challenges associated with ageing on a global scale [66–68]. However, it is essential to carefully select collaborators based on their distinct skills, thereby ensuring that partnerships are formed with complementary strengths [69].

Facility-based interventions played a significant role in strengthening GBV prevention and survivor support by integrating response mechanisms into healthcare settings. Initiatives such as Sexual Assault Referral Centres (SARCs) in Nigeria and therapeutic counselling for IPV survivors in antenatal care (ANC) settings provided structured support to GBV victims. SARCs were successful in improving access to forensic examination, post-exposure prophylaxis, trauma counselling, and legal services, yet gaps remained in their capacity to deliver sustained tertiary prevention services. Survivors often faced difficulties in navigating legal follow-ups, while resource constraints hindered the ability of healthcare providers to offer long-term psychosocial support. Similarly, the ANC-based interventions demonstrated promise in addressing IPV during pregnancy, yet issues such as confidentiality concerns and provider workload limited full implementation. These findings underscore the importance of continued institutional capacity-building, ensuring that healthcare facilities have the necessary infrastructure and trained personnel to provide holistic GBV services. Comparable models have integrated legal, social support and psychological services for IPV survivors, providing more accessible justice mechanisms [70, 71]. However, studies indicate that underreporting and fear continue to hinder survivors from fully utilizing such facilities [70–72].

Our findings also highlight the growing role of media and technology-driven interventions in GBV prevention and response. Programs such as the Text4Life mobile reporting system in Nigeria demonstrated the potential of digital solutions in improving reporting mechanisms and case management for GBV survivors. The integration of mobile technology allowed survivors to access services remotely while engaging civil society organizations and healthcare providers in real-time support. Film-based education initiatives in Senegal similarly proved effective in shifting social norms, with adolescent girls and young women demonstrating improved knowledge of IPV, child marriage, and gender equity. However, technological accessibility remained a challenge in remote areas, where connectivity issues and digital literacy impacted uptake. Additionally, some survivors expressed reluctance to report cases through mobile platforms due to privacy concerns or fear of retaliation. While these interventions show promise, they require complementary strategies such as in-person outreach to ensure accessibility and survivor protection. Technology-driven interventions have also been adopted in several settings, such as mobile apps and helplines, which provide immediate access to support networks, particularly for mental health/psychological support services [73, 74]. While such digital strategies have increased reporting rates, studies highlight that they must be integrated into broader service networks to ensure survivors receive comprehensive protection and support [73, 74].

Unlike previous reviews [75, 76] that used the RESPECT Framework to classify intervention approaches, our study applied the framework to describe the intervention outcomes reported across the included studies. Awolaran and colleagues identified interventions spanning five of the seven RESPECT domains [75], while Kerr-Wilson et al. included interventions across all seven domains [76]. These were broader reviews that targeted global, low-and middle-income countries compared to our study, which reflects a more targeted scope, West Africa. Similar to our findings, intervention outcomes were reported across all seven domains, with the most common outcomes related to access to essential services, shifts in attitudes and norms, and strengthened relationship skills. The narrow focus of our study may explain the limited coverage of outcome domains such as economic empowerment and safe environments that were least reported. This outcome-focused approach provides a complementary perspective to earlier reviews [75, 76] and highlights the range of impacts achieved by GBV interventions implemented in West Africa.

Our study revealed that economic empowerment strategies and their role in reducing IPV were reported in the reviewed studies, finding mixed success in achieving meaningful behavior change. Initiatives such as unconditional cash transfers and National Health Insurance Scheme (NHIS) premium waivers in Ghana aimed to reduce financial dependency and increase women’s agency in household decision-making. While poverty alleviation measures contributed to improved emotional well-being and reduced household conflict, financial empowerment alone was insufficient in dismantling entrenched gender hierarchies. Many women continued to experience IPV despite economic improvements, underscoring the need for interventions that integrate financial assistance with gender-transformative programming, legal protections, and male engagement strategies. Without addressing the structural inequalities that perpetuate violence, economic empowerment efforts may struggle to achieve long-term IPV reduction. A systematic review has shown that similar approaches have been implemented mostly in America with few studies reporting this approach in Africa and Asia [77]. Cash-based incentive programs have shown mixed evidence but have demonstrated reductions in the incidence of suicide and violence against women, children and youth [77]. However, studies indicate that economic interventions must be accompanied by structural policy changes and legal enforcement to achieve a sustainable impact [77].

Finally, our findings suggest that sustainability challenges continue to hinder the long-term impact of GBV interventions, particularly regarding funding limitations, inconsistent implementation fidelity, and survivor follow-up. Programs such as SARCs and COMBAT faced obstacles in retaining trained personnel and maintaining service continuity beyond the initial intervention period. Additionally, tertiary prevention services, such as legal aid, psychosocial counselling, and survivor reintegration, were often underfunded or absent, reducing the overall effectiveness of GBV response frameworks. Similar sustainability concerns have been observed where GBV collaborative efforts faced difficulties in maintaining long-term engagement due to financial constraints and policy fragmentation [72]. Strengthening multi-sectoral collaboration, continued funding, and adaptive programming will be essential in ensuring GBV interventions remain impactful beyond their initial implementation phase.

One major limitation of this scoping review is the exclusive reliance on published studies, which may introduce biases and restrict the comprehensiveness of the findings. Relying solely on published works excludes community-level reports, government documents, and unpublished program evaluations, which may provide valuable insights into locally driven interventions and policy implementation challenges. Many grassroots organizations and local service providers document their experiences in internal reports or case studies that do not make it into academic literature, yet their contributions to GBV prevention are critical. The absence of these perspectives may result in an incomplete understanding of the practical challenges, implementation gaps, and lived experiences of survivors in various intervention settings. To address this limitation, future research should incorporate field reports, program evaluations, and expert consultations documented at the grassroots to provide a more balanced and comprehensive assessment of GBV intervention successes and challenges in different Nations. Combining academic research with locally sourced data can strengthen evidence-based policymaking and ensure that interventions reflect both theoretical effectiveness and practical realities. We acknowledge that clinical trial registries were not included in our search strategy and the exclusion of articles not published in English. This may have led to the omission of ongoing intervention studies and relevant studies published in other languages like French, which is dominantly spoken in West Africa. While our focus was on published and accessible evaluations, future reviews should incorporate trial registries and articles published in other languages to capture a broader spectrum of evidence, particularly for emerging or pilot interventions.

## Conclusion

The fight against gender-based violence in West Africa relies on a multi-sectoral approach. Healthcare providers, community leaders, government agencies, civil society organizations, and international funders collaborate to implement, support, and sustain interventions that promote survivor protection, gender equity, and legal enforcement. There is a need to strengthen institutional support and capacity, ensure consistent training among healthcare providers in GBV units/centres, and have a broader community engagement by increasing outreach and advocacy to change gender norm narratives, as well as address economic vulnerability. These can help bridge existing health service gaps and ensure survivors receive comprehensive assistance in a manner that respects their dignity and autonomy. Our study highlights the limited availability of published evidence on school-based GBV interventions in West Africa, emphasizing the need for further research and program development in this area for a more balanced and integrated approach. This will also help to protect vulnerable populations in educational contexts. Strengthening collaboration and sustainability across these sectors is important in maintaining progress and ensuring continued protection for vulnerable populations.

## Supplementary Information

Supplementary Material 1.

Supplementary Material 2.

## Data Availability

No datasets were generated or analysed during the current study.

## Abbreviations

GBV: Gender-based violence
IPV: Intimate partner violence
FGM/C: Female genital mutilation/cutting
SARCs: Sexual assault referral centres
SRH: Sexual and reproductive health
SGBV: Sexual gender-based violence
UNICEF: United nations children’s fund
IDP: Internally displaced persons
PCC: Person-centred communication
GDG: Gender dialogue groups
MOE: Ministry of education
MOJ: Ministry of justice
NHIS: National health insurance scheme
MWASD: Ministry of women affairs and social development
SRGBV: School-related gender-based violence
DHS: Demographic and health survey
PTSD: Post-traumatic stress disorder
OSF: Open Science framework
UNFPA: United nations population fund
WHO: World health organization
VSLA: Village savings and loans associations
COMBAT: Community action team
ANC: Antenatal care
MOH: Ministry of health
MCN: Mercy corps Nigeria
CSOs: Civil society organizations
SSAGE: Sibling support for adolescents in emergencies
WHARC: Women’s health and action research centre

## Footnotes

1 United Nations geoscheme - Wikipedia. List of countries and territories by the United Nations geoscheme - Wikipedia.
