# Supplementary material for: A scoping review of interventions addressing gender-based violence in West Africa: examining typologies, delivery mechanisms, outcomes, and stakeholder involvement
Source: Confl Health. 2025 Oct 10;19:70. doi: 10.1186/s13031-025-00712-x (PMC12512839; doi:10.1186/s13031-025-00712-x)
Supplement: Supplementary file 1 — Supplementary Material 1. [file 13031_2025_712_MOESM1_ESM.docx]

## Supplementary File One

## **Concepts**

Table 1: Scoping review concepts used to formulate the search strings

| **Concept 1** | **Concept 2** | **Concept 3** |
| --- | --- | --- |
| **Gender based violence** | **Interventions** | **West Africa** |
| Sexual violence  Sexual abuse  Rape  Intimate partner violence  Domestic violence  Female genital mutilation  Sexual assault  Violence Against Women and Girls (VAWG) | Healthcare initiatives  Counseling services  Health prevention programs  School-health programs  Health education program  Community-based initiatives  Awareness campaign | Benin, Burkina Faso, Cabo Verde, Ivory Coast, Gambia, Ghana, Guinea, Guinea-Bissau, Liberia, Mali, Mauritania, Niger, Nigeria, Senegal, Sierra Leone, Togo, |

### Query strings

Search terms using **concepts 1 and 3**

("Gender based violence" OR "sexual violence and abuse" OR "intimate partner violence" OR "domestic violence" OR "female genital mutilation" OR "victims of sexual assault" OR "violence against women" OR "VAWG") AND (“West Africa” OR “Benin” OR “Burkina faso” OR “Cabo Verde” OR “Ivory Coast” OR “Gambia” OR “Ghana” OR “Guinea” OR “Guinea-Bissau” OR “Liberia” OR “Mali” OR “Mauritania” OR “Niger” OR “Nigeria” OR “Senegal” OR “Sierra Leone” OR “Togo”)

Search terms using **concepts 1, 2 and 3**

("Gender based violence" OR "sexual violence and abuse" OR "intimate partner violence" OR "domestic violence" OR "female genital mutilation" OR "victims of sexual assault" OR "violence against women" OR "VAWG") AND (“intervention” OR “Healthcare initiatives” OR “Counseling program” OR “strategy” OR “health response” OR "community-based initiatives" OR "education program" OR “school-health programs” OR "awareness campaign") AND (“West Africa” OR “Benin” OR “Burkina faso” OR “Cabo Verde” OR “Ivory Coast” OR “Gambia” OR “Ghana” OR “Guinea” OR “Guinea-Bissau” OR “Liberia” OR “Mali” OR “Mauritania” OR “Niger” OR “Nigeria” OR “Senegal” OR “Sierra Leone” OR “Togo”)

***Table 2: Inclusion and exclusion criteria for identification and screening of articles and reports***

|  | **Inclusion Criteria** | **Exclusion Criteria** |
| --- | --- | --- |
| **Type of intervention** | Interventions focused on the following forms of GBV:  Sexual violence and abuse  Rape  Intimate partner violence (physical, sexual, and emotional violence)  Domestic violence  Female genital mutilation | Interventions focusing exclusively on child abuse - those below 10 years, human trafficking, or any other forms of GBV that fall outside the specific forms of GBV under the inclusion criteria |
| ***Scope*** | Focused on identifying existing GBV-related health interventions (prevention/ management/ treatment programs), which include  Government health programs  Non-governmental organization (NGO) health projects  Community and school- based health initiatives  Health facility-based services | Studies that reported only non-health related interventions (addressing abuses such political abuse or economic abuse etc) |
| ***Population*** | Women and Men who are in their adult or adolescent age (10 years and above) from different socio-economic groups. Adolescent age group will be determined by the WHO criteria for this age group which is 10 -19 years | GBV interventions implemented for individuals under the age of 10 will not be included |
| **Source** | Peer-reviewed both primary data and analysis of secondary data  Non-peer reviewed documents, technical reports | Not applicable |
| **Language** | Articles and reports only written in the English Language | Articles and reports not published in English Language |
| **Date** | Published between January 2010 and December 2024. This will ensure that all interventions implemented to address GBV in West Africa for two decades are captured. | Studies published before 2010 |
| ***Context*** | GBV interventions implemented in West Africa | GBV interventions implemented in outside West Africa |
