# Supplementary material for: A scoping review of interventions addressing gender-based violence in West Africa: examining typologies, delivery mechanisms, outcomes, and stakeholder involvement
Source: Confl Health. 2025 Oct 10;19:70. doi: 10.1186/s13031-025-00712-x (PMC12512839; doi:10.1186/s13031-025-00712-x)
Supplement: Supplementary file 2 — Supplementary Material 2. [file 13031_2025_712_MOESM2_ESM.docx]

## Supplementary File Two

*Table 3: Findings from the reviewed/ included articles in the study*

| Reference | Geographic location | GBV being addressed | Target group | Mode of delivery | Outcomes |
| --- | --- | --- | --- | --- | --- |
| John et al 2022 [27] | Two urban and two semi-urban local government areas (LGAs), Nigeria | Prevention of Intimate partner violence (IPV) among couples/ cohabiting sexual partners - Physical, sexual, and emotional violence against intimate partners | (Couples) Women aged 18–35 years and their co-residing spouses or long-term partners living in selected communities | Community-based intervention  The training intervention consisted of a package of three interventions—  i) gender socialisation (GS) training,  ii) financial literacy education and  iii) contraceptive counselling services.  Training sessions focused on building knowledge, awareness, and critical consciousness around power, care work, and gender inequalities. | **Relationship skills strengthened** - Improved communication, conflict management, and mutual respect among couples.  Reduction of physical and emotional IPV in intervention arms compared to the control arm.  No significant reduction in sexual IPV across the three arms. |
| Koris et al 2022 [28] | Two urban communities of internally displaced people (IDPs), Nigeria | Prevention of physical violence experienced among adolescent girls - | Male and female caregivers of adolescent girls  Adolescent girls aged 10–14 years. An older male sibling between the ages of 15–19. | Community-based intervention on sibling Support for Adolescents in Emergencies (SSAGE)  Age group reflection and dialogue  One week of training community mentors - Four male and four female respected and dynamic community members as mentors/ facilitators.  Once per week, continued support and training of community mentors by the program implementers  Mentors led separate interactive sessions for adolescent girls, male siblings, and male and female caregivers of adolescents | **Relationship skills strengthened** – improved communication amongst family members due to continued reflection  **Transformed attitudes, beliefs, and norms**  **Violence against adolescents is prevented**  - Greater understanding of gendered power differentials  Decreased perpetration of violence within households.  Data also show instances where unequal power relations across gender and age hierarchies within households persisted. Familial hierarchies that uphold male dominance were maintained |
| Akor et al 2019 [42] | An urban area. The antenatal unit (ANC) of the University of Abuja Teaching Hospital (UATH), Gwagwalada, Nigeria. | Prevention of intimate partner violence (IPV) in pregnancy | ANC women who are victims of IPV | Facility-based intervention (tertiary hospital)  Therapeutic counselling services among women attending ANC  The intervention arm then went through three biweekly counselling sessions, which were made to coincide with their routine visits. 1) **nondirective counselling**, which aims at encouraging the client to discuss her problems with the counsellor who, through listening, affirms the patient’s worth and allows her to take time to express their thoughts; and 2) **problem-solving therapy**, which involves systematically teaching generic skills in active problem-solving to reduce stress and enhance self-efficacy. | **Services enhanced and ensured –** counselling services provided and individualized problem-specific counselling were found to have a statistically significant improvement in their total family function and its dimensions (F. Communication, F. Difficulty, F. Support). In the control arm, none of the dimensions of family functioning (Family Communication, Family Difficulty, and Family Support) demonstrated any statistically significant change at the end of the study. |
| Okonofua, et al 2024 [41] | State level – in rural areas: low-resource settings - 300 communities from 10 (out of 37 total) states in Nigeria's different regions. | Prevention of all forms of GBV, particularly against women and girls | Women and girls | Text4Life community-based intervention  Tele-communication - Use of mobile phones for reporting and managing GBV cases  Capacity-building workshops were held to train the Implementing Partners, Civil Society Organizations, and PHC workers in project implementation.  Awareness creation of the reporting mobile phone channel - Sensitization was carried out through town hall meetings, posters, radio and TV messages, and social media channels. The PHC workers also disseminated information about the device during antenatal visits and immunization clinic days.  Capacity building of PHC workers to provide GBV support services (including counselling), documentation, and follow-up actions such as judicial intervention | **Environment made safe -**  Increased awareness of the reporting channel  Increased reporting of GBV cases with the highest reported being domestic violence and rape.  Improved GBV response and follow-up services |
| Shaw et al 2023 [33] | **Rural and peri-urban** communities in Nigeria.  Low-resource settings | Prevention of IPV, but was called sexual gender-based violence (SGBV) in the article | Young couples | Community-based intervention  Small Group discussions/ community dialogue - reflections on gender equity, SGBV, child spacing, and family planning services.  Use of IEC materials to sensitize community and religious leaders on issues of gender equity, SGBV, and FP. The intervention aimed to increase the uptake of family planning (FP) methods, promote more gender-equitable roles (i.e., childcare and household chores), and reduce the prevalence of IPV among women. | **Relationship skills strengthened** – improved communication among couples due to continued reflection  **Transformed attitudes, beliefs, and norms** - The intervention led to equitable and violence-free relationships among young couples  Reduction in reports of IPV (emotional, physical, and sexual violence) among women at post-evaluation |
| Bamigboye et al 2023 [49] | Not specified whether implemented in urban or rural areas. This was conducted in 2 tertiary healthcare facilities, 14 secondary healthcare facilities, and 11 primary healthcare centres (PHC) | Management of IPV victims | Nurse-midwives working in the maternity units of selected health facilities | Facility-based intervention  **Training of those who delivered the intervention** – researchers & healthcare providers who are in academia  **Capacity-building sessions with nurse-midwives at the healthcare facilities** included lectures, audio-visual aids, and group discussions among participants. At the intervention sites, healthcare providers were trained on how to provide counselling services for IPV clients either on visits or requests via mobile telephone calls. | **Services enhanced and ensured** - Improved knowledge and skills in IPV screening/ case identification and management among healthcare providers  Observed practice of IPV detection and management improved significantly from 21.9 % satisfactory practice before the intervention to 63.5 % after the intervention (p = 0.001) in the experimental group, with no appreciable improvement in practice detected in the control group (21.9% versus 36.5%; p = 0.682). |
| Strengthening the Response to SGBV in Nigeria[51] | State-level implementation -supported 10 sexual assault referral centres (SARCs) across 9 States) | **Strengthening the Response to SGBV** by supporting 10 existing Sexual Assault Referral Centers (SARCs) in nine states across Nigeria. | Health providers at the SARCs | Facility and community-based intervention - Evidence to Action (E2A) Project  Multifaceted intervention: i) Capacity building healthcare providers to improve SGBV service delivery; ii) Strengthening the Referral and Linkage System; iii) Building capacity and support for SARC outreach and community engagement; iv) Public SGBV awareness campaigns using radio and television; v) Operational capacity building and development of a national reporting tool for monthly aggregated data to relevant ministries on SARC services provided; vi) Mentorship and periodic supportive supervision of SARCs in service delivery, programming, documentation, M&E, and finance and grant management; vii) Capacity building for external engagement and coordination | **Services enhanced and ensured** - Improved SARC Organizational Capacity including financial management and M&E capacity areas  Improved Capacity for Service Delivery (strengthen referral services etc)  Improved Capacity to Solicit, Manage, and Administer Additional Funds |
| Strengthening the Response to SGBV in Nigeria[51]  E2A project report fully documented evidence of the previous project (which was not implemented by their project), which their project leveraged to strengthen the capacity of SARCs. | State-level implementation -  Eight SARCs are located in public tertiary or secondary health facilities, and two are free-standing, independent centres | Management of **SGBV** | Nigeria - SARC program/project targeted support services for GBV victims | Facility and community-based intervention (tertiary/ secondary/ private hospital)  **Primary prevention** **strategy**: i) SGBV sensitization programs in schools and communities (involving traditional/ religious leaders), ii) awareness campaigns using information, education, and communication (IEC) materials, radio, and television.  **Secondary prevention** **strategy**: Establishment of 10 sexual assault response centres (SARCs). The SARC provided SGBV survivors with comprehensive services including treatment of injuries, forensic examination, pregnancy testing and emergency contraception, HIV testing services, post-exposure prophylaxis (PEP) for HIV, STI prophylaxis, and trauma counselling by trained experts.  **Tertiary prevention** **strategy**: Although some SARCs provide a few tertiary prevention activities, the SARCs rarely refer clients to tertiary services. These typically include safe homes, education services, psychosocial services, skills acquisition services, and other medical services, such as psychiatry services | **Services enhanced and ensured** -  Established SARCs  The major reported outcome of the project is the establishment of sexual assault referral centres (SARCs)  SARCs, typically embedded within a health facility, provide essential services for SGBV survivors. |
| Abdelnabi et al 2014 [47] | Rural areas/ Low resource settings in four districts, Ghana | Prevention of IPV | Pregnant women attending Group ANC | Facility-based intervention through Basic ANC services and Group education on healthy relationships with IP  The model uses strategies such as storytelling, peer support, demonstration, and teach-back to enhance its effectiveness. Health literacy is incorporated as an integral part of clinical practice within the model, not as an add-on to care.  Training/ capacity building of healthcare workers to deliver the intervention strategies | **Services enhanced and ensured** -  Improved support services  There were no differences in rates of physical, sexual, or emotional violence between groups at baseline however, authors noted small increase in changes in reported emotional violence (from no to yes) in the intervention group compared to the control group (6.2% vs. 4.6%) and sexual violence in the intervention group compared to the control group (5.4% vs. 3.2%). |
| Addo-Lartey et al 2019 [32]  Hornuvo et al 2022 [37]  Ferrari et al 2022 [31]  Ogum Alangea et al 2020 [44]  Stern et al [29] | Rural, peri-urban and urban communities in four districts, Ghana. | Prevention of violence against women and girls | Primary targets are the voluntary community-based (action teams) healthcare workers and secondary targets are the community members. | Community-based intervention   - Training - A six-person Community Action Team (COMBAT) - Awareness on gender-based violence (GBV) by the trained COMBAT through public meetings, in churches, during community festivals, association or group meetings and through radio programs using various strategies such as role plays. - Provision of GBV support services such as referral services and follow-up services for cases reported to service providers - Training of other stakeholders/state agencies such as COMBAT and establishing linkages - Annual refresher training for COMBAT and staff of the selected state agencies | **Services enhanced and ensured** - Improved capacity of COMBAT. Reduced incidence of Violence against women (physical, sexual, and depression)  **Transformed attitudes, beliefs and norms** - Addressed gender attitudes (women’s subordination, tolerance of VAW, VAW perpetration, and controlling behaviours), social stigma for the victims of VAW, institutional response to VAW, relevant laws on VAW, women and societal responses to VAW and mental health issues.  **Strengthened relationship skills -**More gender-equitable roles and decision-making in relationships. Improved intimate relationship skills and quality - enabled reflection and self-expression/ communication  **Safe/ enabling environments** due to the engagement of community leaders/ influencers. Acceptability of intervention by community members |
| Barrington et al, 2022 [30]  Peterman et al 2022 [48] | Predominantly Rural communities in two districts, Ghana. | Prevention of IPV (physical, sexual, and emotional violence)- particularly against women and girls | Women of reproductive age > = 18 years. i) Pregnant women or lactating mothers (child under the age of 12 months), ii) orphans/ vulnerable children, and iii) persons with severe disability who live in households that meet poverty-related criteria. | Community-based intervention  1. Bimonthly unconditional cash payments  2. Waived healthcare service fees through the premiums NHIS  Provides bimonthly cash transfers and premium waivers to enroll households into the National Health Insurance Scheme (NHIS) for women who are pregnant or have a child under the age of 12 months and who live in households that meet poverty-related criteria. | **Economic empowerment and poverty reduction -** reduced intra-household conflict and increased empowerment. Cash transfers did not fundamentally change gender norms or reduce gender-role strain in a context of ongoing economic insecurity, which could limit the gender transformative potential and sustainability of IPV reductions. - Barrington et al, 2022 [30]  No impacts on the experience of 12-month IPV on average using conventional thresholds for statistical significance, but all coefficients are consistently negative. - Peterman et al, 2022 [48]  **Service enhanced and ensured** - While health insurance increased access to healthcare, local norms, shame, fear, and minimal provider screening deterred IPV disclosure to healthcare providers - Barrington et al, 2022 [30] |
| Rohn et al 2023 [34] | National-level implementation (urban and rural settings) in Ghana | Prevention and provision of support services for victims of all forms of GBV | Community members and victims of GBV | Community-facility-based intervention  1. Free treatment services, including counselling services  2. Prevention services through awareness creation at homes, in schools, churches, and markets.  3. Referral services - The Unit also refers victims for medical services and specialized help to clinical psychologists, social workers from the Department of Social Welfare, and counsellors attached to the Unit.  4. Collaboration with NGOs and other CSOs to address the needs of victims | **Service enhanced and ensured** - This study did not evaluate the impact of the intervention on whether it increased or reduced the reporting rate of GBV cases.    But the analysis showed barriers such as a lack of trust in formal support channels, limited awareness of legal resources like support units for victims of violence, and fear of partner arrest prevented women from fully utilizing available services.  From an institutional perspective, barriers ranged from inadequate resources, including administrative and logistical support, to inadequately trained personnel, visibility and distance to support units/centres offices, lack of privacy at the centres, and financial constraints. |
| Kaburi and Kaburi 2023 [45] | Urban community in Ghana | Support services for domestic violence victims | All victims of GBV | Community-facility-based intervention  i)services provided at the healthcare facility and ii) how the police - law enforcement department handles the cases  Established domestic and sexual assault centres that provide a walk-in service where victims and witnesses can report incidents of abuse directly.  The centres also have a dedicated phone line linked to police and hospital facilities across the country as part of the Ghana Police Service’s crisis response intervention. It provides victims with a referral system that includes medical, legal, and counselling services | **Services enhanced and assured** –  Increased provision of services for victims  Survivors became knowledgeable of their supporting services, namely legal services, temporary shelter, and psychosocial support.  Some survivors are revictimized in the process of accessing formal services.  Survivors expect the government to provide them with shelter, upkeep, medical, and legal aid. |
| Silverman et al 2023 [43] | Rural areas - 48 villages selected from three districts, Niger. | Reduction of IPV | Reaching married adolescents (RMA) | Community-based intervention  Small group sessions - Selected female and male community members served as “mentors” and were trained to facilitate small, populated sex-disaggregated groups for married adolescent girls (twice monthly) and their husbands (once monthly).  Individual household visits - monthly visits  Community dialogues - Community dialogues were convened by two trained facilitators each month at the village level, engaging community gatekeepers and key influencers | **Services enhanced and ensured -** Increased demand for modern contraceptive use among non-pregnant women and decreased experience of IPV; Women participating in the RMA intervention were more than twice as likely to report modern contraceptive use at follow-up relative to those in the control arm  Only the intervention arm combining both household visits and small group sessions was effective at both increasing current modern contraceptive use and decreasing past year IPV |
| Ndavi et al, 2024 [64] | Cross-country project, Guinea | Ending FGM medicalisation - where health workers are actively involved in FGM | Health workers and ANC clients | **Facility-based intervention**  Awareness creation and Counselling services during ANC | **Services enhanced and ensured –** Clients reported satisfaction with the discussions  The intervention significantly enhanced providers’ ability to deliver FGM prevention messaging  Impact assessments highlighted shifts in knowledge, attitudes, and reported intentions among both providers and clients, indicating the potential for healthcare-based interventions to play a significant role in preventing FGM/C. |
| Koris et al 2023 [65] | Cross country project, Rural -Refugee Community and Host Communities in Niger | Prevention of physical and verbal violence perpetrated by male siblings and caregivers among adolescent girls | Male and female caregivers of adolescent girls  Adolescent girls aged 10–14 years. An older male sibling between the ages of 13 and 24 | Sibling Support for Adolescents in Emergencies (SSAGE) Community-based intervention  Age group reflection and dialogue  The approach was reported to have been implemented within families by enrolling an adolescent girl along with one of her male siblings and their male and female caregivers into the program. It was stated that synchronized age- and gender-specific sessions were conducted with each cohort over three months, while intra-familial discussions on weekly topics were encouraged throughout the program's duration. | **Relationship skills strengthened** – improved communication amongst family members due to continued reflection  **Transformed attitudes, beliefs, and norms**  **Violence against adolescents is prevented**  (1) decreased reported perpetration of corporal and verbal violence by male siblings, (2) improved perceptions of equity in the division of household labor between male and female siblings, and (3) increased emotional involvement and social support from male siblings to their sisters. |
|  |  |  |  |  |  |
| Nordmann et al 2022 [66] | Both urban and rural areas in the Liberian counties | Case identification and management of FGM/C | Healthcare providers engaged in healthcare provision to FGM/C survivors and were willing to provide informed consent. | Facility-based intervention  Training workshop for healthcare providers – A series of six training workshops based on the WHO handbook Care of Girls and Women Living with Female Genital Mutilation. These workshops targeted nurses, midwives, physician assistants, and traditional midwives across three Liberian counties  Mobile App for Learning and Decision Support – Some workshops introduced an app built with KoBoToolbox®, designed to: Facilitate self-learning about FGM/C management, and also help users self-assess clinical decisions using decision-aid algorithms from the WHO handbook | **Service ensured –**  The study found that the mLearning app for FGM/C survivor care was acceptable, feasible, and useful for healthcare providers (HCPs) in Liberia. The intervention led to several key outcomes, including improvements in knowledge, decision-making in service provision, and awareness about FGM/C.  HCPs viewed the app as a valuable tool for self-learning, clinical decision-making, and follow-up care for FGM/C survivors.  Enhance healthcare providers' competence in managing FGM/C-related complications. |
| le Port et al 2022 [52] | The intervention was implemented in rural areas (120 villages) in Senegal | Reduction of GBV by increasing knowledge, awareness, and improving attitudes | The intervention was targeted at adolescent girls and young women aged 14 to 34 years | Community-based intervention  Filming and discussion after watching  4 monthly workshops organized using the pedagogical kit (discussions include family violence, IPV, work-place violence, forced/ child marriage, FGM/C, rape, sexual assault, reporting to law enforcement, gender equity etc)  Training of facilitators | **Transformed attitudes, beliefs, and norms**  **Violence against adolescents is prevented**  Media-driven awareness campaigns helped in shifting harmful norms limiting access to services. |
| Hossain et al 2014 [67] | Implemented in rural areas - Twelve villages in Côte d'Ivoire | Reduction of GBV | targeted Men | Community-based intervention  Group discussion among men  Training of facilitators of the group discussion  Aimed to shift men’s attitudes from basic awareness about the impact and consequences of violence against women and girls to practicing and trialling behaviour change. The Men’s Discussion Groups offered participating men the opportunity to reflect on new attitudes and practice new behaviours within a supportive environment and to encourage social change within an intimate relationship. | **Transformed attitudes, beliefs, and norms**  Decrease in women’s experiences of physical and sexual IPV, although this trend was not statistically significant.  A lower prevalence of men’s reported intention to commit physical IPV  Increased levels of men who believe a woman has the right to refuse sex under all circumstances |
| Gupta et al 2013 [68] | Rural villages in Côte d'Ivoire | Reduction of physical and/or sexual IPV | Women and their male partners  Eligible Ivorian women (18+ years, no prior experience with group savings) were invited to participate. Women and their male partners | Community-based intervention  Gender Dialogue Group discussion  The intervention participants received both Village Savings and Loans Associations (VSLA) support and an 8-session Gender Dialogue Group (GDG) intervention, structured around the Stages of Change from the Transtheoretical Model. The GDG sessions, designed for women and their male partners, aimed to address household gender inequities. | **Economic empowerment and poverty reduction** - Less likely to report economic abuse  **Transformed attitudes, beliefs, and norms** - Lower odds of reporting physical and/or sexual IPV. The acceptance of wife-beating significantly decreased  However, attitudes toward a woman’s ability to refuse sex did not significantly change. |
| Reilly 2014 [69] | Urban and rural communities in Sierra Leone, | Reduce SRGBV in schools - working to reduce physical violence, sexual violence, and abuse, and increasing the reporting of violence in schools. | Primarily, the adolescent boys and girls, the schools, and the community members | School-community-based intervention  Action strategies  Awareness creation  Community, district, and national level campaigns  Capacity building of school personnel and parents of school children  Establishment of clubs in schools - peace clubs, girls’ rights clubs, and literacy and debating clubs  Production and distribution of IEC materials | **Environment made safe -** Increased awareness of violence and code of conduct among community members and school students/personnel  Increased reporting of violence in schools  Reduction of physical violence, sexual violence, and abuse  Awareness-raising at the community level on issues about girls’ rights and gender-based violence |
|  |  |  |  |  |  |
